# Staff and patient experience of the implementation and delivery of a virtual health care home monitoring service for COVID-19 in Melbourne, Australia

**DOI:** 10.1186/s12913-022-08173-1

**Published:** 2022-07-13

**Authors:** R. L. Jessup, N. Awad, A. Beauchamp, C. Bramston, D. Campbell, Al Semciw, N. Tully, A. M. Fabri, J. Hayes, S. Hull, A. C. Clarke

**Affiliations:** 1 Hospital Without Walls Directorate, Northern Health, 185 Cooper Street, Epping, Melbourne, 3075 Australia; 2 Allied Health Research, Northern Health, 185 Cooper Street, Epping, Melbourne, 3075 Australia; 3 School of Allied Health, Human Services and Sport, La Trobe University, Bundoora, Melbourne, 3086 Australia; 4 School of Rural Health, Monash University, Sargeant St, Warragul, 3820 Australia; 5 Department of Medicine - Western Health, The University of Melbourne, St Albans, Victoria Australia; 6 Faculty of Art, Design and Architecture, Monash University, Clayton, Victoria Australia

**Keywords:** COVID-19, Home monitoring, Implementation science, Patient experience, Staff experience

## Abstract

**Background:**

Provision of virtual health care (VHC) home monitoring for patients who are experiencing mild to moderate COVID-19 illness is emerging as a central strategy for reducing pressure on acute health systems. Understanding the enablers and challenges in implementation and delivery of these programs is important for future implementation and re-design. The aim of this study was to explore the perspectives of staff involved with the implementation and delivery, and the experience of patients managed by, a VHC monitoring service in Melbourne, Australia during the COVID-19 pandemic.

**Methods:**

A descriptive qualitative approach informed by naturalist inquiry was used. Staff interviews were analysed using the Consolidated Framework for Implementation Research (CFIR). Patient experience was captured using a survey and descriptive statistics were used to describe categorical responses while content analysis was used to analyse free text responses as they related to the CFIR. Finally, data from the interviews and patient experience were triangulated to see if patient experience validated data from staff interviews.

**Results:**

All 15 staff were interviewed, and 271 patients were surveyed (42%). A total of four final overarching themes emerged: service implementation enablers, service delivery benefits for patients, fragmentation of care, and workforce strengths. 19 subthemes aligned with 18 CFIR constructs from staff and patient data.

**Conclusion:**

Rapid implementation was enabled through shared resources, dividing implementation tasks between senior personnel, engaging furloughed healthcare staff in design and delivery, and having a flexible approach that allowed for ongoing improvements. Benefits for patients included early identification of COVID-19 deterioration, as well as provision of accurate and trustworthy information to isolate safely at home. The main challenges were the multiple agencies involved in patient monitoring, which may be addressed in the future by attributing responsibility for monitoring to a single agency.

## Background

The coronavirus pandemic (COVID-19) has placed unprecedented pressure on health systems. This has required providers to trial, develop and evaluate new ways of responding to demand. COVID-19 is a mild viral illness for most patients (~ 85%) but may cause severe pneumonitis/pneumonia and death in a small percentage of cases [1]. Provision of virtual health care (VHC) home monitoring for patients who are experiencing mild to moderate illness is emerging as a central strategy for diverting pressure away from acute health systems [2–6].

VHC COVID-19 home monitoring, where in-person appointments are substituted with telephone or video consultations, may more efficiently use limited clinical resources, reduce exposure risk for health care workers, and improve acute health service capacity to accommodate those with more severe illness [5]. Randomised trials of VHC delivery for chronic diseases have reported high satisfaction rates amongst both patients and staff, with comparable clinical and service outcomes [7].

Many VHC-based models of home monitoring of COVID-19 have now emerged internationally, including models that monitor biometrics using technology, and others that use interactive chatbots [8–13]. However, there is limited evidence available on the perspectives of staff involved with the implementation and delivery of VHC COVID-19 home monitoring, or the experience of patients managed by these services. Addressing this gap will add to the community health and implementation science literature to assist with re-design and help inform implementation of these models in the future. The aim of this study is to describe the experience of staff and patients involved with a rapidly implemented VHC home monitoring service for COVID-19 and to elucidate any key learnings using the Consolidated Framework for Implementation Research.

## Methods

We used a descriptive qualitative approach informed by naturalistic inquiry [14, 15]. We chose this methodology as we sought to provide an accurate observation, through narrative description, of the experiences of staff and patients.

We used two different qualitative approaches to data collection. Staff were interviewed to gain insight into their experience of service implementation and delivery, including enablers and challenges. Patients were surveyed as we wanted to capture from a broad sample to gain insight into satisfaction with the care received. We triangulated the qualitative data collected from the staff interviews and patient surveys to determine whether staff perception about the value for patients was validated by patient reported experience.

### Setting and context

In Australia, the largest outbreak of COVID-19 in 2020 was in Melbourne, accounting for 75% of all cases (*n* = 20,336 on 24^th^ October 2020), and 90% of all deaths (*n* = 817) [16]. In response to rising COVID-19 case numbers, a state of emergency was declared in Victoria on the 16^th^ of March 2020 which remained in place until the 8^th^ of November 2020 [17]. The number of daily incident cases in Victoria in this first wave of the pandemic peaked on the 5th of August [18].

Northern Health (NH) is the key provider of acute public health care in the northern region of Melbourne, Australia. Residents living in the catchment are culturally and linguistically diverse, originating from over 180 countries, and speaking over 106 different languages [19]. Much of the catchment experiences greater disadvantage than state averages [20], and it has been disproportionately impacted by the COVID-19 pandemic; while it accounts for approximately 10% of Victoria’s population, at the peak of the pandemic in August 2020 one third of COVID-19 cases resided in the catchment [21, 22].

The home monitoring service was a rapidly developed VHC model where telephone contact was provided by trained health professionals to patients with COVID-19 who were self-isolating at home. Isolating at home meant that individuals were not permitted to leave the home for any reason except to seek medical care. The VHC home monitoring service was proposed on the 15th of July 2020 and was implemented on the 21st of July 2020, approximately 4 months after the commencement of the outbreak. Clinical staff, predominantly nurses and allied health staff, who had been furloughed due to medical risk issues, were engaged to work in the service. Policies and procedures were developed rapidly, initially based on a procedure that was shared by another health service that had already established a home monitoring service. This procedure was adapted to fit the local context, approved by local infection control teams, and refined over the first four weeks of the service.

Patients were monitored by phone during the acute phase of their illness which was typically up to 14 days. Unlike home monitoring models that incorporated remote monitoring of temperature, pulse rates and oxygen saturation, this was a low technology service that did not include biometrics. All patients with COVID-19 in the Northeast of Melbourne were managed on this service and were referred following a positive PCR result by the Victorian Department of Health (DoH). The DoH is the central health system and aged care funding body for Victoria. Participation in the home monitoring service was optional but highly recommended, and approximately 98% of COVID-19 positive patients agreed to be enrolled in the service. A clinical assessment using a script, including a checklist of risk factors that fed into a risk stratification (Table 1), was used for all patients on entry into the program. Social and welfare needs assessment were also undertaken, and patients experiencing issues such as domestic violence or drug, suicidal ideation or drug and alcohol concerns were referred to the hospital social work and/ or psychology service for immediate care and referral if required.

**Table 1:** Community monitoring service risk stratification

High Risk	Any of: • Age 60 or over • Presence of one or more co-morbidities associated with increased mortality (cardiovascular disease, chronic lung conditions, hypertension, diabetes mellitus, cancer, chronic kidney disease, obesity) • Immunosuppression • Aboriginal or Torres Strait Islander • Pregnant women • Socially isolated / vulnerable (including individuals who are psychosocially complex or have limited self-management skills) • Frailty • A person discharged after an acute inpatient admission at NH (this does not include patients discharged directly from the Emergency Department) • A person who has had a 000 call due to COVID-19 symptoms • Moderate to severe COVID-19 symptoms • A person whom clinical judgement/clinician worry identifies at being at higher risk (e.g. shortness of breath associated with infection)
Low Risk	• Under 60 years of age • No co-morbidities • Nil known immunosuppression • Mild COVID-19 symptoms

Patients stratified as at risk of deterioration were offered ongoing monitoring via a daily phone call; those at lower risk were offered second daily phone calls or SMS. In follow up phone calls, patient symptoms were categorised as stable or deteriorating. For patients with deteriorating symptoms, escalation of care involved either consultation with a medical officer, referral to the general practitioner or hospital emergency department, or calling an ambulance. An analysis of outcomes for the service has been published previously [23].

The service did not have the ability to issue clearance from isolation for individuals to return to normal activities – this could only be issued by the Victorian DoH. While the health care network provided health and support monitoring for COVID-19, the DoH concurrently conducted compliance monitoring of COVID-19 positive cases, so many patients experienced phone contact from both the hospital home monitoring service and the DoH.

### Ethics approval

This study was approved by the NH Human Research Low Risk Ethics Committee (reference number 68253).

## Measures

### Staff experience

Staff experience was measured using semi-structured interviews. All interviews were conducted by the same researcher (RLJ, female, PhD qualified) who was experienced in conducting qualitative interviews and who was working as the Allied Health Research Lead at the hospital and was not involved with the implementation or delivery of the service. An interview guide was used with question areas based on the Consolidated Framework for Implementation Research (CFIR). The CFIR is a theoretical framework for implementation research considered well-suited to health service studies. The CFIR assesses both the effectiveness of implementation within one context and the factors that might affect implementation within other contexts [24]. The CFIR contains 39 constructs grouped within five domains: 1) Intervention characteristics, 2) Outer setting, 3) Inner setting, 4) Characteristics of individuals, and 5) Process. The interview guide was developed by RLJ and reviewed by all AB and AC. The guide was piloted with three individuals external to the research team. No changes were made to the guide following the pilot and these interviews were not transcribed or included in the data analysis. The guide and associated domains and constructs are provided in Table 2.

**Table 2:** Staff Interview guide

Question Prompts	CFIR Domain & Construct/s
Can you describe your role in the COVID-19 Community Monitoring Service?	Process; all constructs
How confident are you that the COVID-19 Community Monitoring Service is responding to individual and community needs during the pandemic? What gives you that level of confidence (or lack of confidence)?	Characteristics of individuals; all constructs
Did the service work for all patients that were approached? Why/ why not?	Process: planning + reflecting and evaluating
Tell me about the supports, materials, or toolkits that were available to help you in your role within the service? How do you access these materials?	Intervention; design quality Outer setting; patient needs & resources
What are the most important benefits that have been achieved with this service? To what extend has the patient/clients’ needs been met? How do you know these are benefits? Have there been any unintended consequences? Can you tell us any stories about the patient experience that stand out for you?	Intervention characteristics; all constructs
Do you believe the majority of the staff on the team are happy with how the service operates? Describe	Characteristics of individuals:;all constructs Inner setting; culture + compatibility
Do you believe the majority of the patients that were provided care were happy with the service? Describe	Intervention characteristics; all constructs
If the COVID-19 pandemic continues at current numbers can this service change continue to be delivered in this format consistently moving forward? Why/why not (Prompt) Does this intervention fit within our health service/ health system? Is it feasible to continue?	Intervention characteristics; adaptability + structural
What kinds of changes or alterations did you need to make to the service to work more effectively (as telehealth delivery/other) as the service has evolved?	Process – executing, reflecting, evaluating

### Patient experience

Consumers were included in the development of an experience survey, which included four questions using a categorical response format, two open ended questions and a single binary question. The first question asked *Please provide an indication of frequency and method of contact by the service* and had the following response options: daily phone call, phone call every second day, mix of phone call and texts, text messages only. Additionally, there were three questions with a Likert scale response option (strongly agree, agree, neither agree nor disagree, disagree, strongly disagree): *I felt that I was able to get the help I needed from the service, I felt supported to understand how to isolate at home, I felt supported to manage my symptoms*. Two open ended questions asked participants: *Can you given an example of advice you received that you found helpfu*l and *Are there any additional comments about your experience with the Coronavirus (COVID-19 Home Monitoring Service) that you would like to share?* The final question asked participants whether they would recommend the service to family and friends if they were to contract COVID-19 (Yes/No).

### Participant recruitment

#### Staff experience

All staff involved in establishing the service and/or providing direct patient telephone support were invited to participate via email and consented to participate verbally over the phone prior to their interview. All fifteen staff participated in the interviews. Interviews were between 17 and 55 min in duration. Five staff were allied health (Physiotherapy, Exercise Physiology and Occupational Therapy discipline), one was medical (Respiratory Physician) and nine were nurses (emergency, paediatrics, cardiac and generalist community and ward trained). Four staff were responsible for development, implementation, and management of the service, four were involved in both the development and implementation as well as provision of service, while the remaining seven staff were part of the direct care team involved only in provision of service (Table 3).

**Table 3:** Staff interview participants

Discipline	Number of participants (*n* = 15)	% female	% direct care team
Allied Health	5	80%	60%
Nursing	9	100%	78%
Medical	1	0%	100%

Participation in the interviews was voluntary, and staff were informed that they could choose to end their participation at any time and could withdraw participation up to two weeks post interview, after which time the interviews were transcribed and de-identified. Interviews were conducted from 15^th^ of September to 29^th^ of November 2020. All interviews were recorded using the Tape-A-Call® platform and were transcribed verbatim.

#### Patient experience

All patients over the age of 18 who were enrolled in the service were invited to provide feedback on their experience of receiving the service via a patient satisfaction survey. A total of 850 patients were enrolled in the service. Due to the speed of implementation, ethics approval was gained after the service had been implemented and therefore only those who were enrolled following approval and who responded to a post discharge phone call (*n* = 646) were eligible to participate. Overall, 271 patients participated in the survey (response rate 42%). Mean age of overall sample was 34 (SD 17) years, 54% were female and 48% were born overseas.

The survey was voluntary and anonymous and participants were informed that no identifiable information would be collected and responses would not impact on future care. Completion of the survey was considered implied consent to participate which was made explicit in the survey preamble. Participants were invited to complete the survey at the end of treatment and were given the option of responding over the phone or via a link sent to their phone or email. All data were collected using REDCap®. Where the patient opted to complete the satisfaction survey over the phone this was completed with the support of a NH staff member that was independent to the service team. Where participants did not speak English, interpreters were used to conduct the survey over the phone. Data was collected from the 12th of September to the 9th of November 2020.

### Analysis

#### Staff experience

**Fig. 1:**
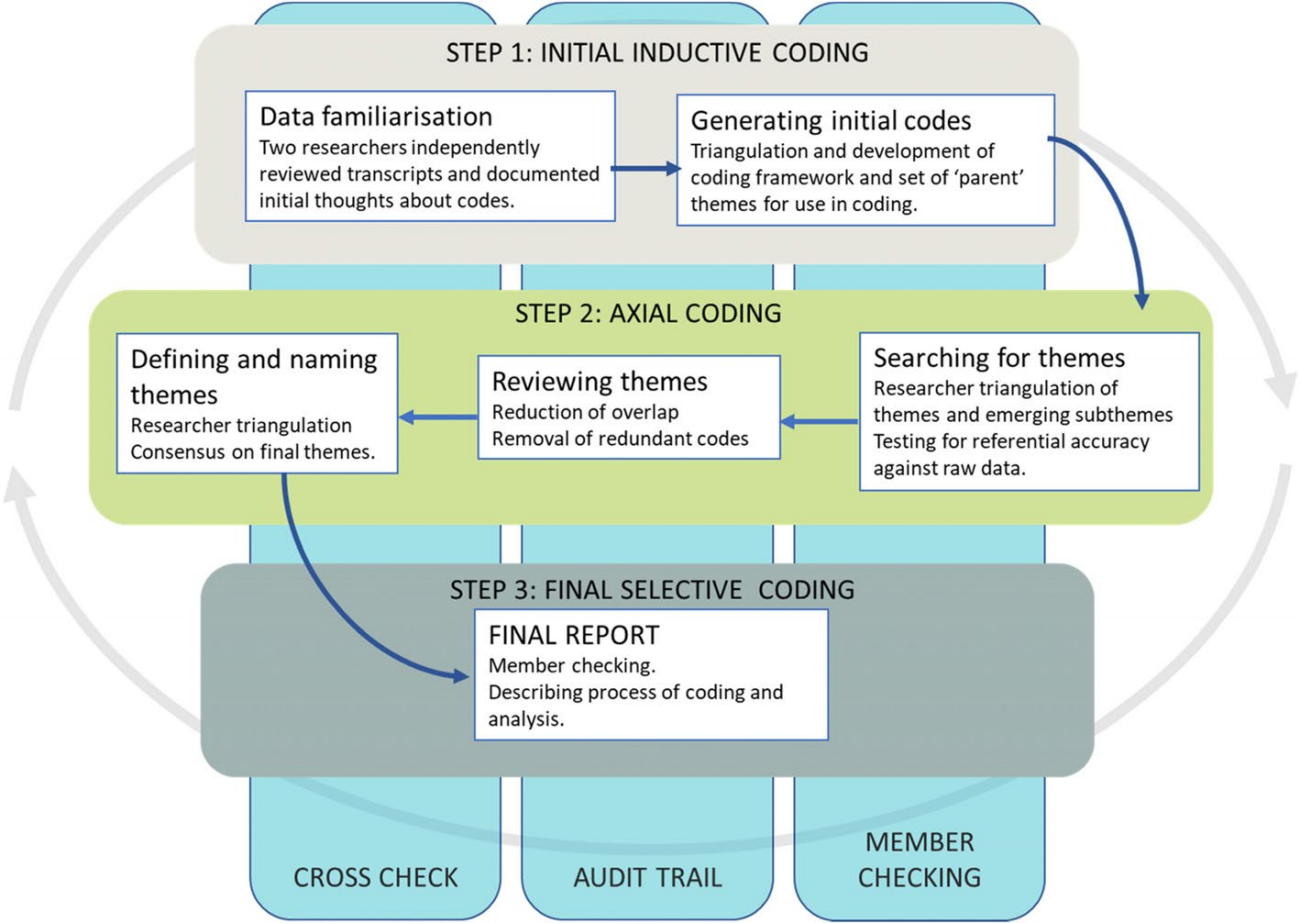
Thematic analysis process

As the CFIR framework can present some limitations when applied to complex adaptive system interventions [25, 26], we did not use the CFIR framework to create pre-defined codes, but conducted a thematic analysis based on Braun and Clarke’s six steps [27]. Figure 1 provides an overview of the thematic analysis process. To reduce subjectivity and bias, and to ensure trustworthiness of final inferences, two researchers (RLJ and NA) independently coded all data [28] in NVivo. An audit trail was kept, and final themes were sent back to interviewees for respondent validation [29, 30]. The coding process involved three steps—an initial inductive open coding step, a second axial coding step to reduce overlap and redundancy of coding, followed by a final selective coding step to identify and refine final themes as they related to the CFIR constructs. Saturation was pre-defined as three consecutive interviews where no new theoretical categories are identified past the initial minimum sample of twelve [31]. A minimum sample size of 12 was identified based on the literature [32–34].

#### Patient experience

For patient experience data, descriptive statistics were used to report categorical, binary and Likert scale responses, with agree and strongly agree response options considered overall agreement with statement for Likert items. Content analysis was used to analyse the two open ended questions. Two reviewers (RLJ and AC) created pre-formulated codes following an initial read through of responses [27]. The same two reviewers then independently applied the coding. Cohen’s Kappa was used to determine inter-rater reliability, with 0.81 – 1.00 considered almost perfect agreement, 0.61 to 0.80 considered substantial agreement, and 0.41 – 0.6 considered moderate agreement [35]. We therefore considered an acceptable level of agreement to be greater than 0.41. Where agreement was less than 0.41, the two reviewers met to compare coding and to see if higher agreement could be achieved.

### Data reporting

A descriptive narration was used to report findings. Consolidated criteria for Reporting Qualitative research (COREQ) guidelines, a validated 32-item checklist for reporting qualitative data, have been followed to ensure completeness of information presented.

## Results

### Staff experience

Across the 15 interviews, thematic saturation was reached. Four themes, with 19 subthemes emerged that aligned with 18 CFIR constructs. Table 4 provides an overview of each of the final themes, CFIR constructs and supporting quotes. These themes are discussed further below.

**Table 4:** Thematic analysis of staff interviews and content analysis from patient surveys

Theme	Sub-theme	Example quote	CFIR Domain	Construct
**THEME 1: Service commissioning enablers**	Command centre/ Division of labour	*‘I played to people’s strengths or areas and used a command centre approach [to service establishment].’ Interview 11*	Inner Setting	Leadership engagement
				Relative priority
	Redeployment of frontline personnel furloughed due to health concerns	*‘We were able to redeploy staff who were pregnant or who had health concerns that would put them at risk if they staying working on the front line.’ – Interview 13*	Inner Setting	Available resources
	Dynamic and flexible approach to change	*‘We had huddles twice a day with the group as well as the leadership team… it was through those huddles we [made] continual changes to that procedure.’ – Interview 14*	Intervention characteristics	Adaptability
	Rapid development of policy and procedures and centralised access	*‘They created a shared drive which the majority of our information went into; introduction packages that we sent to patients [*etc.*]. The policy itself is on Prompt [hospital intranet], templates we used when speaking with patients, so that its consistent … was emailed … and was on the [shared] drive so you could access it yourself, and as they got updated, they emailed all of us so that way if there were any changes we knew straight away.’ – Interview 1*	Outer Setting	Patient needs and resources
			Inner Setting	Networks and communications + Available resources + Access to Knowledge & information
**THEME 1: Service commissioning challenges**	Inadequate staffing initially to meet demand	*‘One of the memories that I have is when we started it was right sort of as that peak was really hotting up and we had three staff at that point.’ – Interview 13*	Inner setting	Readiness for implementation
	Sporadic commencement of staff	*‘We had staff starting on different days. This meant I kept being taken away from the call centre to train the new staff when we were really busy. This could be improved by having staff all start on the same day.’—Interview 4*	Process	Planning
**THEME 2: Service delivery perceived benefits for patients**	Managing deterioration	*‘When I called to talk to him his wife answered the phone and she said he can’t talk at the moment, he’s really sick and I’m trying to get him to the hospital, can I talk to you later on. I said no I can actually help you, do you need some help? She explained the situation … that she was trying to get him to hospital and she couldn't. I offered to speak to her husband and managed to have bit of a conversation with him and built a rapport. I built up enough trust with him that he then let me call an ambulance for him.’ – Interview 1*	Characteristics of individuals	Knowledge and Beliefs about the Intervention
	Support to self-isolate safely and reduce household transmission	*‘We were providing … advice … around how to isolate safely at home away from other people, like good hand hygiene, separation from other members of the household, when and wear a mask, how to safely move about the house to reduce the risk of household spread.’ – Interview 2*	Characteristics of individuals	Self-efficacy
		*The service was the best service- because we had no friends or family support, you gave us good advice on how to isolate to prevent the spread of the virus- Patient survey respondent 168*		
	Welfare checks	*‘I had one patient that I’d been following up every day for a good 4 or 5 days and one of the days that I rang her, probably about 15 min later than normal, she said “I’ve been waiting for your to call. You make my day.”’ – Interview 6*	Characteristics of individuals	Individual stage of change
	Provision of information and clarification	*‘There’s a lot of people who don’t know what to do. Information is very limited so even when we tell them to do this and that, sometimes they would get surprised and go “oh I can go out” and I say no because you’re a close contact of this patient so basically you need to be home as well until that patient is cleared. There was some confusion…’ – Interview 7*	Characteristics of individuals	Other personal attributes
	Information provided in language	*‘We focused on [people] who don’t speak English and got a person … to interpret. Sometimes one of the family members interprets and that is not appropriate so we provided telephone interpreting services and [translators] locally through Northern Health. That worked very well.’ – Interview 1*	Characteristics of individuals	Access to knowledge and information
	Improved co-ordination of care and patient flow	*‘We would call the emergency department if the patient was coming in just to let them know that a Covid* + *patient was coming in.’ – Interview 4*	Process	Engaging + Executing
**THEME 3: Fragmentation of care**	Navigating multiple systems	*‘[There was] a gap between us and the department [DHS] …we have no [ability to provide] clearance so the patient was still hanging on between us.’ – Interview 8*	Outer setting	External policy and incentives
	Disjointed care leading to delays and reduced quality of care	*‘People who were in isolation for a long time had secondary respiratory issues. From what I understood they [DHS] have a very binary metric or if you’re still symptomatic you’re going to stay in isolation….they wouldn’t then go and do anything about that in terms of ‘ok lets get one of our doctors to come out and assess you or get you back to ED and figure out what’s going on with you’. One patient had over 40 days of isolation. We had to fight for him.’ – interview 2*	Outer setting	External policy and incentives
	Single point of contact for patients would improve care	*‘If it was to happen again I think each healthcare service should be responsible for their local area but there would need to be better co-ordination between health services.’* *– Interview 2*	Outer setting	External policy and incentives
		*‘Thankyou for the daily phone calls to see how my husband was… but there were too many phone calls everyday from "everyone"- Patient survey respondent 23*		
**THEME 4:Workforce strengths**	Mix of disciplines	*‘The ED and ICU guys understand that acute medical deterioration, but then people like physios and other allied health who work in the community understand the broader contextual needs from a social wellbeing point of view or access.’—Interview 2*	Inner setting	Implementation climate
	Meaningful work	*‘I think it goes to that idea of people having meaningful work, and I am important’ – Interview 4*	Inner setting	Implementation climate
	Peer support	*‘Initially I was very hesitant to work here because I’ve worked in ED for almost 10 years and I hate change but because ED is not safe for me at the moment, I was offered… I mean they wanted me to get redeployed in this job and initially I thought oh my god, I don’t know I can do it. From day one they have been welcoming and I didn’t get intimidated at all because my suggestions were always welcome, they would always listen and stuff so yeah I’m just…I’m thankful that I have been redeployed here.’ – Interview 7*	Inner setting	Learning climate

### Theme 1: service implementation enablers and challenges

#### Inner setting: leadership engagement

Leadership engagement was considered essential to the success of this service, all the way from the hospital executive to the leadership within the team itself. The senior executive team at NH requested a home monitoring service be implemented within a week. To help facilitate this rapid establishment, key implementation tasks were divided up across four senior personnel within the team, referred to in the interviews as a ‘command centre’. This ‘command centre’ division of labour was allocated across the following: workforce, telecommunication, patient management systems and policies and procedures. Telephone systems were set up in a call centre style. with a central phone number that patients could ring to access support. Patient management systems were established to track patients and ensure reporting capability.

#### Inner setting: relative priority

The potential risks to both the individual (management of deterioration) and the community (spread of infection) created a sense of urgency and the service was given high priority within the organisation. The DoH provided a specific funding stream to the health service to support establishment of the service.

#### Inner setting: available resources

One of the key success factors for rapid implementation was being able to draw on existing staff as a resource. Frontline healthcare staff with pre-existing medical conditions who had been furloughed due to exposure risk were approached and redeployed into the direct care team.

#### Inner setting: readiness for implementation/ process: planning

Rapid implementation requirements meant that initial staffing levels were still not adequate to meet the demands of the service. Staff engaged early reported feeling overwhelmed by the volume of patients. Those responsible for training new staff reported feeling frustrated by having been removed from monitoring patients to onboard new staff at irregular intervals. The service would have benefited from more planning time, and onboarding staff in a consistent manner.

#### Outer setting: patient needs & resources/ inner setting network and communications, available resources and access to knowledge and information

This service benefited from access to existing resources in the form of written policy and procedures that had been developed by another hospital as a basis for their service. This was adapted for use locally. Telephone scripts were developed to ensure consistency of approach, and SMS links were provided to trustworthy information about important aspects of self-care, including identification of deterioration, were created that could be sent through to patients.

#### Intervention characteristics: adaptability

The first four weeks were crucial to the success of the service, with staff having to apply a dynamic and flexible approach to manage constant change, reorganisation, and progress over time. During these first weeks, the service adapted in response to feedback from patients and staff working in the service, with additional features added. This included text messages as an alternative to telephone calls for those at low risk of developing complications.

### Theme 2: service delivery benefits for patient

#### Characteristics of individuals: knowledge and beliefs about the intervention

In all 15 interviews, the main reported strength of the service was its ability to adapt to provide personalised support and education for patients. This allowed staff to build rapport and trust. In some cases, staff reported that this was what made all the difference when it came to convincing a patient that they required an ambulance review or hospital attendance.

#### Characteristics of individuals: self-efficacy, other personal attributes and access to knowledge and information

Staff reported that initially they were tasked with symptom and welfare checking, with the primary aim of early detection of deterioration. However, it quickly became evident that an information vacuum existed and that many enrolled patients were struggling to find information about how to safely manage COVID-19 in the home. Staff reported that they regularly responded to questions about how to safely isolate one family member within a large household, including how to manage used crockery and cutlery, shared bathrooms and interaction within the home. Having access to interpreters to assist with translating information for patients from migrant backgrounds was essential, and staff often had to provide clarification of misinformation in other languages. Overall, staff reported that supporting large households to understand isolation requirements played a crucial role in reducing community transmission of the virus.

#### Process: Engaging and executing

One of the reported benefits of running a home monitoring service within the hospital setting was the ability for improved co-ordination of care. When staff identified that a patient was deteriorating and required a hospital attendance, they were able to contact the emergency department to provide a handover. Staff also had ready access to infectious disease specialists and other important specialists for high-risk patients (e.g., obstetricians). Patient management and clinical systems were also linked, so medical staff managing deterioration had access to information about the patients’ health over the preceding days.

### Theme 3: fragmentation of care

#### Outer setting: external policy and incentives

Consistently reported across all interviews, the greatest challenge experienced by staff was the fragmentation of services and division of roles between the healthcare network (NH) and the centralised services at DoH. Staff reported that patients (and sometimes even the staff themselves) struggled to understand the difference between the work being carried out by the two agencies, or to recognise who was contacting them, and some reported that they felt burdened by the number of contacts they received.

The DoH also monitored patients’ symptoms with the purpose of assessing their COVID-19 status and for providing clearance for them to return to normal activities. NH were unable to provide this clearance for patients, however, staff were able to act in an advocacy role when patients failed to meet the criteria for clearance.

Finally, a number of staff also reported that duplication of care could also occur across health services, with a number of health services potentially being involved with a single household, depending on the test site for individual members. Staff reported that an approach that allowed health care providers within geographical areas to provide all monitoring services would be an improvement if similar services were operationalised in the future.

### Theme 4: workforce strengths

#### Inner setting: implementation climate

Rapid implementation of this service was made possible by the ready access to experienced and knowledgeable clinically qualified staff who had been furloughed from other active clinical roles within the health service. These staff brought a variety of skills and abilities and complemented one another. The staff mix included medical (respiratory physician), allied health (physiotherapy and exercise physiology) and nursing (paediatric, emergency and palliative care). Staff identified that this skill mix meant there was always someone in the team who could answer a question if another team member was uncertain.

The team reported a strong sense of pride about their ‘contribution to the war effort’. Working in the home monitoring service felt like a privilege when they were unable to work in their normal roles and still wanted to be able to contribute to the management of the pandemic.

#### Inner setting: learning climate

All staff that were interviewed reported they felt well supported by their peers in the service and by the leadership team, and that they were given opportunities to contribute to the development and the direction of the service (including through improving the telephone scripts, and policies and procedures). The rapid change in workforce roles meant some reported feeling nervous initially.

### Patient experience

**Fig. 2:**
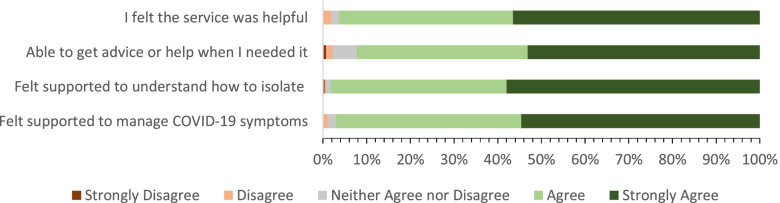
Patient reported experience of care

Most participants who responded to the survey received phone calls either daily or second daily (Table 5). Overall, surveyed patients were highly satisfied with the care they received (Fig. 2). 96% of surveyed patients felt the service was helpful (*n* = 261), 92% felt that they were able to get the help they needed from the service (*n* = 250), 98% felt supported to understand how to isolate at home (*n* = 266) and 97% felt supported to manage their symptoms (*n* = 263). In response to the question would you recommend the service to a friend or family member if they had COVID, 236 (87%) of respondents said that they would.

**Table 5:** Method of contact with Home Monitoring Service for patients participating in the survey

Type of contact	No. of responses (%)
Daily phone call	107 (39)
Phone call every second day	67 (25)
Mix of phone calls and texts	53 (20)
Text messages only	20 (7)
Did not respond to this question	24 (9)

The two open ended questions were coded across 19 predefined themes. These themes, the number of respondents and the inter-tester reliability for the initial coding and re-coding are provided in Table 6. Only two themes had a poor level of agreement (below 0.41) and were recoded. When discussed, 100% agreement was reached.

**Table 6:** Themes and inter-rater reliability for content analysis of free text responses

Content analysis	n	%	Cohen's Kappa first round coding
***Responses to 'Can you given an example of advice you received that you found helpful?' n* = *230***			
General advice was helpful about COVID-19	18	8%	0.74
Advice on how to monitor my health	17	7%	0.5
Advice on how to reduce household transmission	7	3%	0.81
Advice on how to isolate at home	23	10%	0.84
Advice and provision of PPE	11	5%	0.87
Advice on how to access to essential supplies	8	3%	0.89
Advice on how to manage my symptoms	8	3%	0.72
***Responses to 'Are there any additional comments about your experience with the community monitoring service that you would like to share?' n* = *186***			
Service was easy to contact / access	10	5%	0.43
Service made too many calls to me	3	2%	0.66
Service was able to answer my questions	16	9%	0.81
Service identified deterioration and helped me	10	5%	0.86
Too much duplication between providers	13	7%	0.33^a^
Service provided me with mental health support	10	5%	0.68
Introduction of SMS option was good	5	3%	0.83
Regular phone calls were helpful	36	19%	0.68
Felt supported by the service	56	30%	0.49
Grateful to have someone to talk to	4	2%	0.56
Welfare check important/ felt cared for	11	6%	0.52
Service facilitated clearance	4	2%	0.33^a^

^a^ the two first round items that were < 0.41 were subsequently 1.0 after discussion

Thirty-one patients chose not to respond to the question on examples of advice and 77 had no comments to provide about the service. The most frequently reported example of advice received was on how to isolate at home (*n* = 23, 12%). The most common open-ended response about experience related to feeling supported by the service (*n* = 56, 30%).

A small number of patient participants (*n* = 13) supported the findings from staff interviews when they identified frustrations associated with fragmentation of care between DoH and the NH service.

Ten (5%) respondents identified that the service recognised deterioriation quickly and managed the transition to hospital for which they were grateful, while a further four (2%) identified that the service facilitated clearance to return to normal activity for them. Overall, the service escalated 30 individuals (3.5% of all) for urgent medical care [23]. These findings support the findings from staff interviews. Additionally, responses initiated by patients demonstrated the importance of advice on how to safely isolate at home (*n* = 23) and how to reduce household transmission (*n* = 7) support the feedback from the staff interviews that this was an important part of the service.

## Discussion

This study provides important learnings from the experience of both staff and patients involved in the rapid implementation and delivery of VHC COVID-19 home monitoring service. Home monitoring services have been established in many countries to provide an important and effective alternative to hospital care for low risk patients with COVID-19. Our study found that while this service provided an essential role in early detection of deterioration, it also ensured that enrolled individuals had adequate access to locally relevant information and supports in order to safely isolate at home and manage their condition.

Internationally, most health systems have implemented a test, trace, isolate and support approach to managing the COVID-19 pandemic [36] Self isolation is a foundation of this strategy, as it reduces transmission and infection rates in communities. However, unlike other methods of containment, the capacity to self- isolate at home requires adequate supports [37]. Access to accurate and trustworthy information about how to manage COVID-19 when isolating at home, and how to protect other family members, was a key issue for patients monitored in the VHC home monitoring service. Early in the pandemic, government agencies and public health experts responsible for communicating information necessarily focused on engaging the public with health protective behaviours [38]. Our findings suggest that once infected, access to clear and accurate information on why, when and how to isolate was not as easy to find. Communications on how to isolate should be clear and tailored to different audiences, and this needs to be coupled with access to sufficient food and medications, and a safe space to isolate [39]. This study demonstrates the importance of VHC home monitoring services in delivering both personalised information and practical supports to support self-isolation.

While health systems internationally differ, all countries have had to respond to the COVID-19 crisis by redistributing finite health resources toward the response. For many, this has included staff being required to join new teams in an unfamiliar role, and changes to their work environment [40]. Our findings suggest that providing staff with a sense of control over decisions around redeployment/ role transition, and seeking staff contribution towards decision making, led to a high sense of satisfaction amongst staff and reduced the risk of transition shock.

We found that the phone calls provided from muliple agencies added to confusion for both the patients and staff involved in the service. The DoH phone calls were not aimed at provision of support but at discouraging unlawful violation of isolation rules. Studies internationally have found that penalties are unlikely to encourage compliance, and that access to adequate income, food and medications, as well as a sense of collective responsibility, are more likely to ensure infected individuals within communities comply with self-isolation requirements [41–43]. In Victoria, public health units managed by hospitals have now been established that aim to provide a single local response and leverage community connections to keep future outbreaks contained [44]. Ensuring that a single agency is responsible for COVID-19 monitoring will reduce fragmentation, improve trust in systems, and improve the experience of care delivery for both staff and patients.

Our previous publication demonstrated that the service was effective in detecting deterioration, and no deaths were recorded [23]. This study adds to these findings in providing further evidence of the value of the service, as well as identifying some of the challenges experienced in rapid implementation. It further reinforces that a low technology, high touch approach provided by skilled clinicians operating a call centre is both effective and highly valued by staff and patients alike. This finding is important for future implementation, particularly in low resource settings where biometric monitoring is not possible. Implementation in other settings would benefit from strong leadership and commitment, shared resources, and a single point of contact for patients to provide support, education and manage clinical deterioration.

### Utility of the CFIR

Our study found that only 18 of 31 potential constructs were represented in the data from the 15 staff interviews. We found it challenging applying the CFIR, with some overlap across constructs for a single theme, while other constructs were not represented in the data at all. This may be due to the type of intervention that was implemented. The home monitoring service was a rapidly implemented, time-limited program for patients experiencing a short-term acute illness. Many of the constructs focus on implementation of services that will be sustained, where an evidence base exists and implementation staff and organisations may compare models and chose one that best fits their needs and available resources. The model described here was previously untested, and will be sustained no longer than the life of the pandemic.

### Strengths and limitations

A strength of this study is the triangulated design with the patient survey responses supporting the findings from staff interviews. However, our study is limited by several considerations. One is that these findings are from a single hospital network in Melbourne and so the results may not be generalisable to other hospital networks or other populations. In addition, due to the lag time between implementation of the service and ethical approval to conduct this study, approximately 2/3rds of the patients were not surveyed about their experience of the service. Although there is no suggestion that the sub sample of surveyed patients was otherwise biased in any way, there is always the possibility that if all had been surveyed the responses may have been different.

## Conclusion

This study suggests that enablers of rapid implementation COVID-19 home monitoring services include sharing resources, engaging a ready-to go workforce, dividing tasks amongst senior personnel and having a flexible approach that allows for ongoing improvements following implementation. Early identification of deterioration and provision of person centred, accurate and trustworthy information were the main benefits of the service. A challenge experienced by both staff and patients was that responsibility for care was divided between multiple agencies (health and welfare provided by the hospital, compliance with government regulations and clearance by DoH). Implications for practice and policy are that future programs should be delivered by a single agency who can manage both deterioration and clearance from isolation. Future research should determine whether the service is effective in reducing hospitalisation and bed-days and ideally should include a cost–benefit analysis.

## Data Availability

De-identified participant data from this research will be shared upon reasonable request with the corresponding author.

## Abbreviations

CFIR: Consolidated Framework for Implementation Research
DoH: Department of Health
NH: Northern Health
SMS: Short Messaging Service
